# Prognostic value of a lactate metabolism gene signature in lung adenocarcinoma and its associations with immune checkpoint blockade therapy response

**DOI:** 10.1097/MD.0000000000039371

**Published:** 2024-10-04

**Authors:** Tengfei Huang, DuoHuang Lian, MengMeng Chen, YaMing Liu, MeiQing Zhang, DeHua Zeng, Shun-Kai Zhou, WenMin Ying

**Affiliations:** a Department of Thoracic and Cardiac Surgery, The 900th Hospital of the Joint Logistics Support Force of the People’s Liberation Army, Fuzhou, Fujian Province, China; b Department of Radiotherapy, Fuding Hospital, Fuding, Fujian Province, China.

**Keywords:** immune checkpoints blockade therapy response, immune infiltration, lactate metabolism, lung adenocarcinoma, prognostic model

## Abstract

Lung adenocarcinoma (LUAD) is a study that examines the prognostic value of lactate metabolism genes in tumor cells, which are associated with clinical prognosis. We analyzed the expression and clinical data for LUAD from The Cancer Genome Atlas database, using the GSE68465 dataset from the Gene Expression Omnibus and the MSigDB database. LASSO Cox regression and stepwise Cox regression were used to identify the optimal lactate metabolism gene signature. Differences in immune infiltration, tumor mutation burden (TMB), and response to immune checkpoint blockade (ICB) therapy were evaluated between groups. LASSO and Cox regression analyses showed an eight-lactate metabolism gene signature for model construction in both TCGA cohort and GSE68465 data, with higher survival outcomes in high-risk groups. The lactate metabolism risk score had an independent prognostic value (hazard ratio: 2.279 [1.652–3.146], *P* < .001). Immune cell infiltration differed between the risk groups, such as CD8^+^ T cells, macrophages, dendritic cells, mast cells, and neutrophils. The high-risk group had higher tumor purity, lower immune and stromal scores, and higher TMB. High-risk samples had high tumor immune dysfunction and exclusion (TIDE) scores and low cytolytic activity (CYT) scores, indicating a poor response to ICB therapy. Similarly, most immune checkpoint molecules, immune inhibitors/stimulators, and major histocompatibility complex (MHC) molecules were highly expressed in the high-risk group. The 8-lactate metabolism gene-based prognostic model predicts patient survival, immune infiltration, and ICB response in patients with LUAD, driving the development of therapeutic strategies to target lactate metabolism.

**Key points**

1. A lactate metabolism gene-based risk prognostic model was established to predict the overall survival of patients with lung adenocarcinoma.
2. The lactate metabolism risk score was an independent prognostic factor from the tumor stage.
3. A high lactate metabolism risk score was associated with high tumor purity and low antitumor immune scores.
4. A high lactate metabolism risk score indicated a poor response to immune checkpoint blockade therapy.

## 1. Introduction

Lung cancer is one of the most common malignant tumors worldwide. Global cancer statistics reveal an estimated 2,206,771 newly diagnosed lung cancer cases in 2020, representing approximately 11.4% of the new global cases.^[1]^ Lung cancer led to approximately 1,796,144 cancer-caused deaths in 2020, representing approximately 18% of deaths caused by cancer worldwide, and was the leading cause of cancer-related deaths.^[1]^ A previous study identified an increasing trend in lung cancer incidence among females and a decreasing mortality trend in both sexes in China from 2000 to 2018, comparing trends with those in the United States.^[2]^ Lung adenocarcinoma (LUAD) is the primary subtype of lung cancer and is usually diagnosed at a later stage.^[3]^ Despite the successes in approved therapies,^[4,5]^ the prognosis of patients with LUAD remains poor with overall survival (OS) of fewer than 5 years.^[6]^ Therefore, it is necessary to identify novel therapeutic targets and develop effective prognostic predictors for patients with LUAD.

One of the hallmarks of cancer is metabolic reprogramming, which increases cancer survival and progression.^[7,8]^ The Warburg effect is common in solid tumors and describes glycolysis-dominated energy metabolism irrespective of aerobic conditions, during which glucose is metabolized to generate lactate.^[9]^ As the major metabolic end-product of glycolysis, lactate has been linked to both the malignant properties of tumor cells and clinical prognosis.^[10–12]^ Lactate can induce the expression of programmed cell death protein ligand 1 through its receptor, GPR81, and inhibition of this receptor in lung cancer cells leads to reduced programmed cell death protein ligand 1 levels and its promoter activity.^[13]^ Reduced intracellular lactate levels can contribute to an anti-cancerous immune microenvironment in lung cancer.^[14]^ Accordingly, enzymes and proteins involved in lactate generation and transportation have been previously investigated as either biomarkers or therapeutic targets. For example, lactate dehydrogenase (LDH) has been proposed as a biomarker for monitoring treatment responses in patients with advanced non-small cell lung cancer (NSCLC).^[15,16]^ A decrease in LDH levels of more than 20% was correlated with a favorable radiological response,^[15]^ while high pretreatment LDH levels were correlated with poor OS and progression-free survival.^[16]^ Inhibition of monocarboxylate transporter 4 decreases lactate export into the extracellular space, increasing intracellular lactate levels, and inducing cell apoptosis, which suggests that monocarboxylate transporter 4 may be a therapeutic target.^[17]^ Perhaps unsurprisingly, treatment with the MCT1 inhibitor AZD3965 suppresses tumor growth, increases dendritic and natural killer cell infiltration in lymphoma,^[18]^ and improves radiosensitivity by decreasing lactate transport in lung cancer.^[19]^ These findings emphasize the importance of lactate metabolism in tumorigenesis and lung cancer treatment.

In this study, we developed a lactate metabolism-related risk score model using the LUAD expression and survival data from The Cancer Genome Atlas (TCGA) database. The lactate metabolism risk score (LRRS) stratified patients with LUAD into groups with different overall survival, immune infiltration status, and identified patients with LUAD who are more sensitive to immune checkpoint blockade (ICB) therapy.

## 2. Methods

### 2.1. Sources and preprocessing for data

We acquired the log2 (FPKM + 1) expression data and clinical information of LUAD in TCGA database. We also downloaded the LUAD microarray dataset GSE68465 in the Gene Expression Omnibus database. After we removed samples without survival time or survival time = 0, 504 samples in the TCGA cohort were retained for analysis, and 442 samples in GSE68465 were selected for validation.

### 2.2. Identifying lactate metabolism-related genes

We searched for lactate metabolism-related genes using the MSigDB (version 7.5.1) database with the search terms “lactic acid” or “lactate.” Five lactate metabolism-related gene sets were selected: GOBP_LACTATE_METABOLIC_PROCESS, HP_INCREASED_SERUM_LACTATE, HP_LACTIC_ACIDOSIS, HP_LACTICACIDURIA, and HP_SEVERE_LACTIC_ACIDOSIS. After removing repeated genes, 220 lactate metabolism genes were chosen for analysis.

### 2.3. Establishment and validation of lactate metabolism-related risk score model

Univariate Cox regression analysis provided in the Survival package (version 2.41-1) was applied to screen prognosis-associated lactate metabolism genes and genes with *P* < .05. The LASSO Cox regression analysis provided in the lars package (version 1.2) determined the best weighting coefficient for prognosis-associated lactate metabolism genes. After 10-fold cross-validation of the penalty parameters, the minimum criterion was determined using the optimal value of the penalty parameter *λ*. Next, the LRRS model was established using stepwise Cox regression provided in the Survminer package (version 0.4.9) with the following equation: Risk score = *h*_0_(*t*) × exp(*β*_1_*X*_1_ + *β*_2_*X*_2_ + ...  + *β*_n_*X*_n_), where *h*_0_(*t*) and *β* refer to the benchmark risk function and regression coefficient, respectively, and *H* (*t*, *X*) is the risk function related to *X*(covariable) at time *t*. LRRS was calculated for each sample to assign samples to high and low LRRS according to the median value. Kaplan–Meier curves were plotted to assess the differences in OS between the 2 LRRS groups. Receiver operating characteristic (ROC) curves were plotted to assess the accuracy of LRRS in predicting 1-, 3-, and 5-year survival.

### 2.4. Assessing independent predictive factors

Univariate and multivariate Cox regression analyses were conducted to investigate the associations of prognosis with LRRS and clinical information, including pathologic_T, pathologic_N, tumor stage, age, and sex. The cutoff value was set at *P* < .05.

### 2.5. Nomogram development

A nomogram was developed using the rms package (version 5.1-2) by combining the LRRS with independent predictive clinicopathological factors. A calibration curve was plotted to validate the nomogram. ROC curves were plotted to assess the accuracy of the nomogram for predicting survival at 1-,3-, and 5-year survival.

### 2.6. Assessing the tumor mutation burden (TMB)

Based on the mutation data in TCGA, the mutation frequency of genes and TMBs were analyzed using the Maftools package (version 2.6.05). Differences in TMB between the different risk groups were analyzed using the Wilcoxon test.

### 2.7. Evaluation of immune infiltration

The relative abundance of 22 tumor-infiltrating immune cells was assessed using CIBERSORT, and immune and stromal scores were assessed using ESTIMATE to infer tumor purity. Major histocompatibility complex (MHC) molecules and immune regulator genes were downloaded from the TISIDB database. Data differences between the 2 LRRS groups were compared using the Wilcoxon test.

### 2.8. Immune checkpoint blockade therapy response and cytolytic activity

The tumor immune dysfunction and exclusion (TIDE) database was used to assess the response to ICB therapy.^[20]^ The immune cytolytic activity (CYT) score was calculated using the log-average expression values of GZMA and PRF1.^[21]^ The Wilcoxon test was applied to compare the differences in TIDE and CYT scores between the 2 LRRS groups.

## 3. Results

### 3.1. Identification prognosis associated lactate metabolism genes for model construction

**Figure 1.**
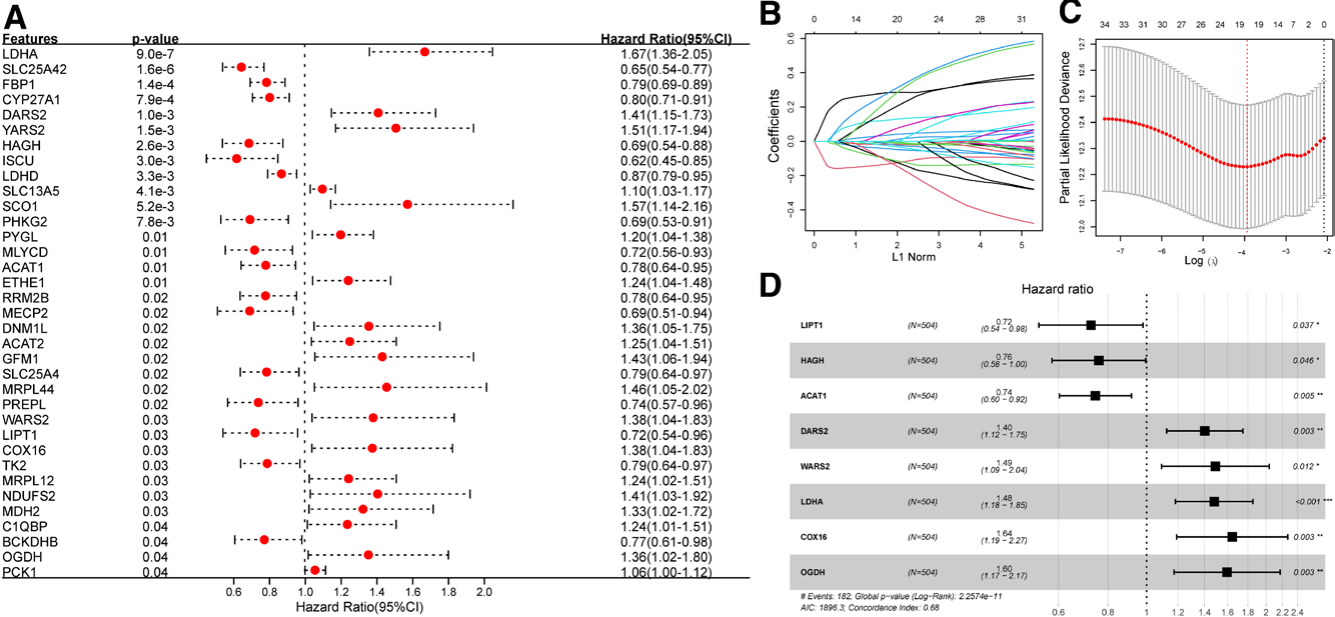
Identification of lactate metabolism gene signatures. (A) Forest plot showing lactate metabolism genes associated with prognosis in univariate Cox regression analysis. (B) LASSO coefficient distribution of lactate metabolism genes. (C) 10-fold cross-validated likelihood deviance of the LASSO coefficient for parameter selection. (D) Forest plot showing the optimal lactate metabolism gene signature in stepwise Cox regression.

From the MSigDB database, 220 lactate metabolism genes were selected for analysis. Univariate Cox regression analysis revealed that 35 lactate metabolism genes were associated with the OS of patients (Fig. 1A). LASSO analysis determined 19 prognosis-associated lactate metabolism genes with the best weighting coefficient (Fig. 1B,C). Stepwise Cox regression identified an optimal 8-gene signature (Fig. 1D). *LIPT1*, *HAGH*, and *ACAT1* were associated with favorable survival outcomes with hazard ratios <1, whereas *DARS2*, *WARS2*, *LDHA*, *COX16*, and *OGDH* were associated with poor survival outcomes (hazard ratio > 1). These 8 genes were selected to establish the LRRS model.

### 3.2. The LRRS is an independent prognostic factor

**Figure 2.**
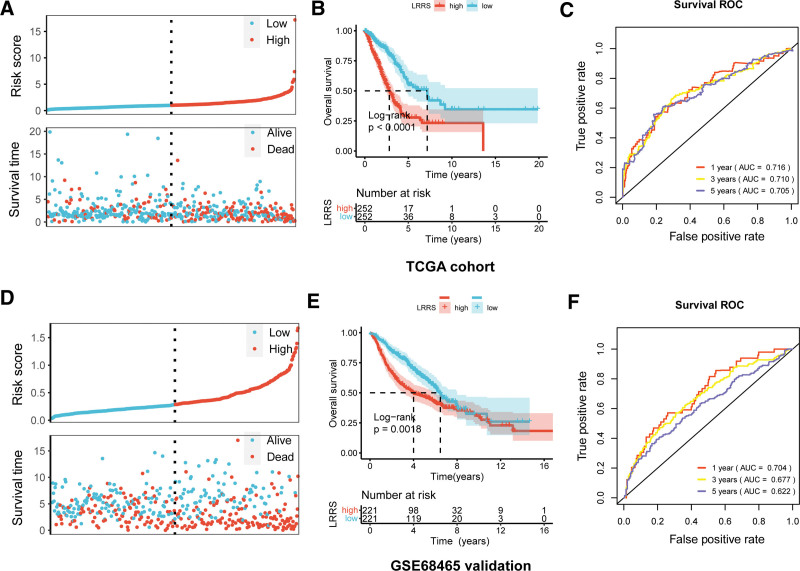
Evaluation and validation for the lactate metabolism risk model. (A–C) Evaluation of the risk model in the TCGA cohort. (D–F) Validation of the risk model using the external GSE68465 dataset. A and D, scatterplots showing the distribution of risk score and the survival status of patients; B and E, Kaplan–Meier curves showing the survival differences in the 2 risk groups; C and F, ROC curves showing the predictive performance for 1-, 3-, and 5-year survival. LRRS = lactate metabolism-related risk score, ROC = receiver operating characteristic, TCGA = The Cancer Genome Atlas.

The LRRS was calculated for every sample to assign each patient to either the high or low LRRS groups (Fig. 2A). Patients with a high LRRS had worse survival outcomes (Fig. 2B). ROC curves revealed that the LRRS showed good reliability with an area under the curve (AUC) of 0.716, 0.710, and 0.706 for 1-, 3-, and 5-year survival, respectively (Fig. 2C). The model was validated using the GSE68465 external dataset and produced consistent results. The LRRS divided patients in the GSE68465 dataset into high and low LRRS groups, and patients with a high LRRS had shorter survival times (Fig. 2D,E). The AUC for 1-, 3-, and 5-year survival were 0.704, 0.677, and 0.622, respectively (Fig. 2F). These findings suggest that the LRRS can be a prognostic factor for patients with LUAD. To further investigate whether the LRRS had independent predictive value, univariate and multivariate Cox regression analyses were conducted in the TCGA cohort (Fig. S1, Supplemental Digital Content, http://links.lww.com/MD/N400). Tumor stage and LRRSs were independent prognostic factors for LUAD.

### 3.3. Assessing a predictive nomogram

**Figure 3.**
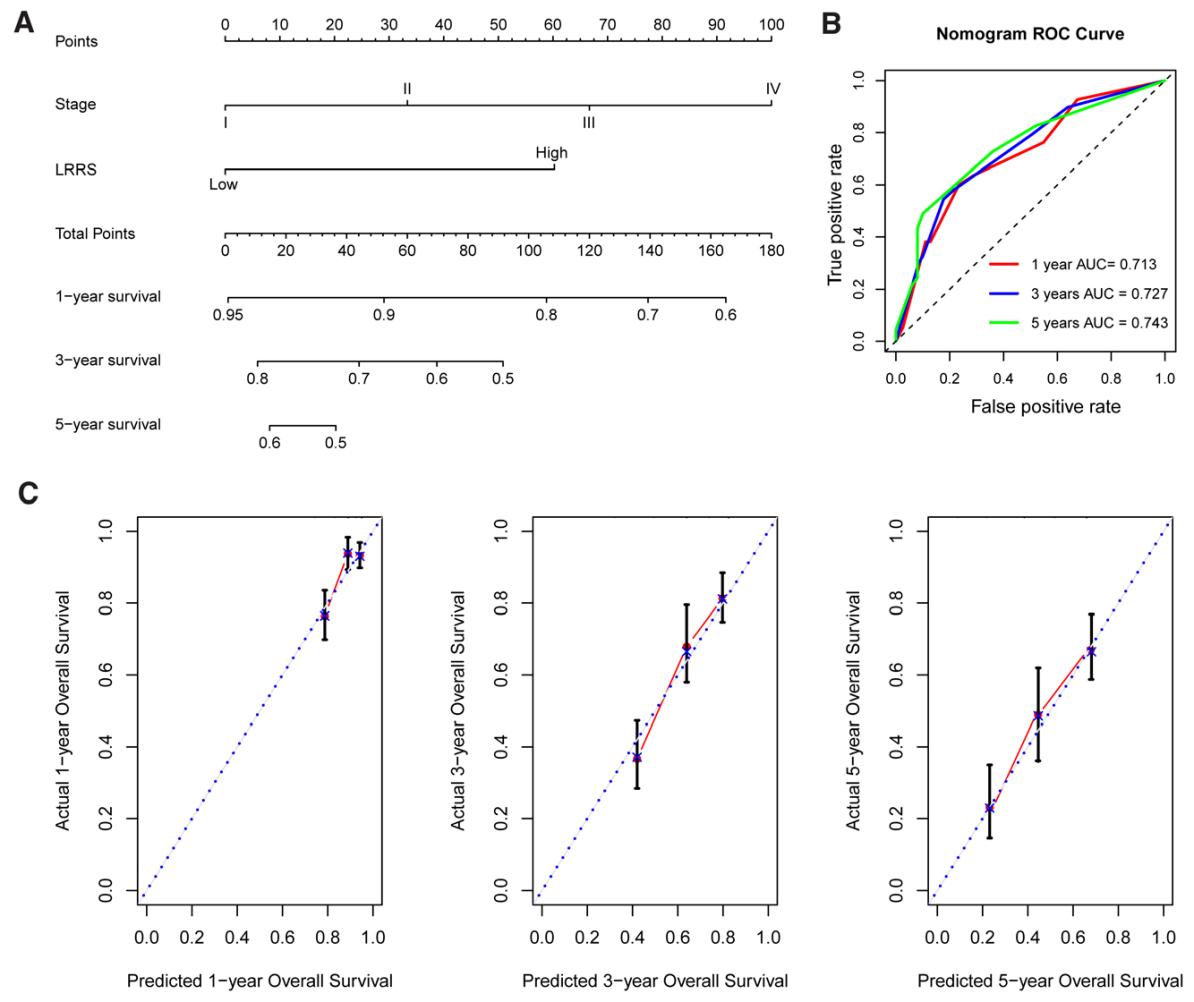
The predictive nomogram for LRRS and tumor stage. (A) Nomogram model established by tumor stage and LRRS. (B) ROC curves showing the predictive performance for 1-, 3-, and 5-year survival. (C) Calibration plot showing the agreement of predictive survival probability with actual 1-, 3-, and 5-year survival. LRRS = lactate metabolism-related risk score, ROC = receiver operating characteristic.

We also established a nomogram based on the 2 independent predictive factors (tumor stage and LRRS) for predicting the probability of survival in patients with LUAD (Fig. 3A). The AUC for the nomogram predicting 1-, 3-, and 5-year survival was 0.713, 0.727, and 0.743, respectively (Fig. 3B). A calibration plot was developed to validate the predictive power of the nomogram, in which the estimated probability approximated the actual survival probability, indicating good predictive power (Fig. 3C).

### 3.4. Identifying associations of the LRRS with TMB and immune infiltration

**Figure 4.**
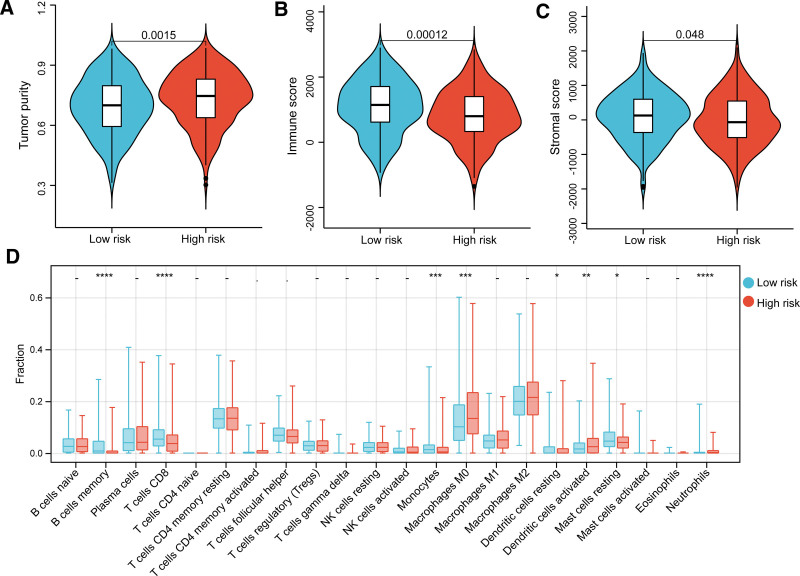
Immune infiltration evaluation between risk groups. Differences in tumor purity (A), immune score (B), and stromal score (C) between the 2 risk groups. (D) Differences in the relative infiltration abundance of 22 immune cells between the 2 risk groups.

Gene mutations in samples from these 2 LRRS groups were characterized (Fig. S2A,B, Supplemental Digital Content, http://links.lww.com/MD/N401). For all samples, missense mutations accounted for the majority of the variation types, and most of the genes were mutated multiple times as indicated by the high proportion of multi-hit variation. Samples in the high LRRS group tended to show a higher mutation frequency than those in the low LRRS group, such as the mutation frequency for *TP53* (53% vs 40%) and *TTN* (47% vs 36%). In summary, samples in the high LRRS group showed higher TMB than those in the low LRRS group (Fig. S2C, Supplemental Digital Content, http://links.lww.com/MD/N401). We also evaluated the immune microenvironments of the samples from the 2 LRRS groups. Samples with high LRRSs had higher tumor purity (Fig. 4A), a lower immune score (Fig. 4B), and a lower stromal score (Fig. 4C), which partly explains the poor survival in the high LRRS group. In addition, we identified the relative infiltration abundance in the tumor microenvironment (TME) of 22 immune cells, and significant differences in 8 immune cells were observed, including memory B cells, CD8^+^ T cells, monocytes, M0 macrophages, resting dendritic cells, activated dendritic cells, resting mast cells, and neutrophils (Fig. 4D). For example, the fraction of memory B cells, CD8^+^ T cells in low LRRS group were significant higher than these in high LRRS group.

### 3.5. Associations of LRRS with ICB therapy

**Figure 5.**
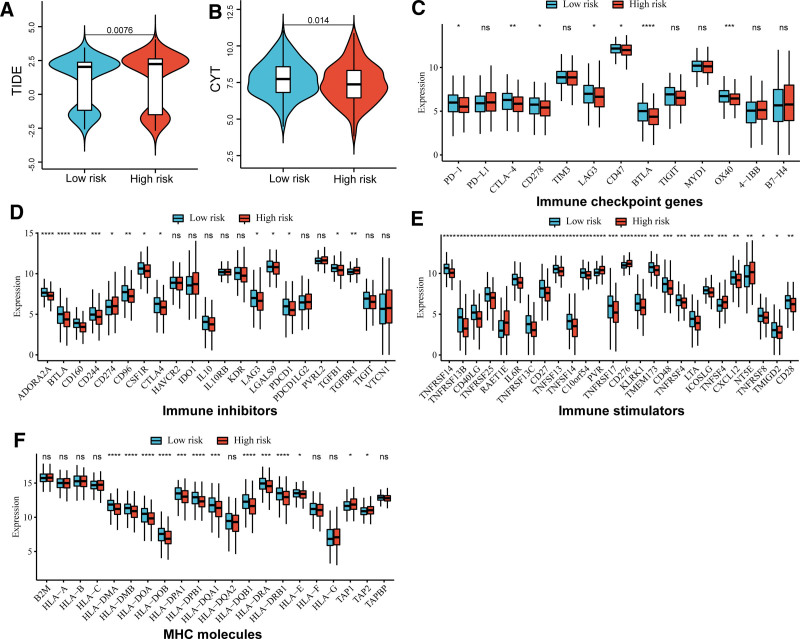
Associations of lactate metabolism risk score with ICB therapy. Boxplot showing the differences in the TIDE score (A), CYT score (B), expression of immune checkpoint molecules (C), immune inhibitors (D), immune stimulators (E), and MHC molecules (F) between the 2 risk groups. ICB = immune checkpoint blockade, MHC = major histocompatibility complex, TIDE = tumor immune dysfunction and exclusion.

To discover any LRRS associations with ICB therapy response, we used the TIDE algorithm to assess the TIDE score. Samples with a high LRRS had high a TIDE score, suggesting a poor response to ICB therapy (Fig. 5A). Previous studies have demonstrated that the CYT score is a biomarker that reflects anti-tumor immune responses and improved prognosis.^[21,22]^ Consistently, samples with low LRRS had higher CYT scores than those with a high LRRS (Fig. 5B). Additionally, significant differences in the expression of several immune checkpoint genes, immune inhibitors, immune stimulators, and MHC molecules were observed (Fig. 5C–F). Most of these genes were lowly expressed in the high LRRS group, such as immune checkpoint *PD-1*, *CTLA-4*, and *BTLA*, immune inhibitors *ADORA2A*, *BTLA*, and *CD160*, immune stimulators *TNFRSF14*, *TNFRSF13B*, *CD40LG*, MHC molecules *HLA-DMA*, *HLA-DMB*, and *HLA-DOA*.

## 4. Discussion

In oncology, it is important to identify gene signatures for predicting prognosis or treatment response based on specific gene sets or hallmarks.^[23–25]^ For instance, Mai et al^[26]^ developed of lactate metabolism-related lncRNA signature as a prognostic model for LUAD, Zhao et al^[27]^ identified a lactic acid metabolism-related gene signature contributes to predicting prognosis, immunotherapy efficacy, and tumor microenvironment of LUAD, and Jiang et al^[28]^ constructed a novel risk score model of lactate metabolism for predicting OS and immune signature in LUAD. However, gene signatures constructed in these studies are not sufficient for immunotherapy of LUAD. The Warburg effect is a major tumor metabolic adaption to favor glycolysis, and the resulting increased lactate levels increase tumorigenesis, metastasis, and antitumor immune inhibition.^[29,30]^ Thus, in this study, we developed a lactate metabolism gene set signature to predict the prognosis of patients with LUAD, and this gene signature was associated with immune infiltration, TMB, and response to ICB therapy.

Of 220 lactate metabolism genes, 35 prognosis-associated genes were identified, and the optimal genes for prognostic prediction were selected; these genes included *LIPT1*, *HAGH*, *ACAT1*, *DARS2*, *WARS2*, *LDHA*, *COX16*, and *OGDH*. *LIPT1* encodes for lipoyltransferase 1, and its functional deficiency can cause lactic acidosis.^[31]^ *HAGH* encodes for glyoxalase II, which is involved in lactate production by converting methylglyoxal into d-lactate under a glutathione catalyst.^[32]^ *ACAT1* encodes for acetyl-CoA acetyltransferase 1, which can stimulate both the Warburg effect and tumor growth by decreasing pyruvate dehydrogenase activity, and it has been proposed as a promising anti-tumor therapeutic target.^[33,34]^ ACAT1 levels are increased in Lewis lung tumor tissue, leading to tumor growth and metastasis, which can be addressed by ACAT1 inhibition by avasimibe, suppressing tumor growth, and enhancing anti-tumor immune response.^[35]^ Aberrant expression of *DARS2* and *WARS2* is associated with lactate elevation^[36]^ and lactic acidosis.^[37]^ In addition, WARS2 was found to be a pro-angiogenic enzyme.^[38]^ Elevated expression of *LDHA* was an independent predictive value for worse survival of patients with LUAD^[39]^ and was linked to radioresistance in patients with NSCLC.^[40]^ Inhibition of LDHA by its inhibitor oxamate markedly enhanced radiation sensitivity and increased autophagy and apoptosis in A549 and H1975 cells.^[40]^ COX16 functions as an inhibitor of p53 activity in various NSCLC cells.^[41]^ *OGDH* encodes mitochondrial 2-oxoglutarate dehydrogenase, and its activation impairs the redox state of A549 cells in a manner similar to p21-dependent cisplatin action.^[42]^ Despite understanding these gene and protein functions, the prognostic value of most genes has not been investigated in LUAD. This will be further investigated in the future.

Using these 8 genes, we developed a lactate metabolism risk score model that stratified patients with LUAD into different risk populations with different survival rates. The LRRS had an independent predictive value for the prognosis of patients with LUAD. Moreover, the nomogram developed using both the LRRS and tumor stage could accurately predict the probability of 1-, 3-, and 5-year survival for patients with LUAD. These findings support the prognostic value of this lactate metabolism gene signature.

Lactate produced in tumor cells is excreted into the extracellular environment through MCT, which leads to acidification of the TME, which in turn establishes an immunosuppressive microenvironment.^[29]^ For example, tumor-excreted lactate can induce tumor-associated macrophage polarization to the tumor-promoting M2 phenotype^[43]^ and repress the cytotoxic activity of natural killer cells.^[44]^ In this study, we investigated the association between lactate metabolism risk score and immune cell infiltration. Our results shown that a high LRRS was associated with high tumor purity and low immune and stromal scores, and the fraction of memory B cells, CD8^+^ T cells in low LRRS group were significant higher than these in high LRRS group, which are major immune effector cells.^[45]^ Our findings suggest low antitumor immunity in patients with a high lactate metabolism risk. We also found that lactate metabolism risk score was associated with infiltration of dendritic cells, mast cells, and neutrophils. Dendritic cells are responsible for capturing and presenting antigens to T cells and have been identified as important regulators of the anti-tumor immune response.^[46]^ Mast cells contribute to the remodeling of the TME by interacting with both tumor cells and infiltrating immune cells, and they also promote angiogenesis and invasiveness by releasing pro-angiogenic factors and matrix metalloproteinases.^[47]^ Tumor-associated neutrophils are myeloid cells that account for a considerable number of pro-inflammatory cells in the TME.^[48]^ In early-stage lung cancer, tumor-associated neutrophils enhance T cell responses but not immunosuppression.^[49]^

We also investigated the association between lactate metabolism risk score and response to ICB therapy, and the results shown that samples with a high LRRS had a high TIDE score, suggesting poor responses to ICB therapy.^[20]^ The TMB has been considered a promising biomarker for immunotherapy, and a high TMB tends to be linked to better results from ICB therapy.^[50]^ In this study, we found that samples in the high LRRS group showed higher TMB than those in the low LRRS group, which seemed to be inconsistent with the TIDE score in this study. However, it has also been reported that a high TMB does not accurately predict ICB therapy response in all solid cancers.^[51]^ This may explain the inconsistency between high TMB and high TIDE scores in the high LRRS group. The CYT score has been proposed as a biomarker to reflect antitumor immune responses and improved prognosis.^[21,22]^ Consistently, we found that samples with a high lactate metabolism risk score had low CYT scores in this current study, indicating poor prognosis and lower anti-tumor immune responses. Numerous studies reported some strategies for cancer immunotherapy, including supramolecular biomaterials, emerging nano-/biotechnology, tannic acid-Fe^3+^ metal-phenolic network (MPN), etc,^[52–59]^ thus it is crucial to further investigate and apply our findings to clinical immunotherapy for LUAD.

This study has some limitations. First, the data were obtained from public databases, and relevant experiments are required. Second, the CIBERSORT method was only used to explore the fractions of infiltrating immune cells, and other tools and flow cytometry are needed to further verify the robustness of the results obtained in this study. Third, the 8 key genes screened in this study should be further tested in other cohorts and through further experimental analyses.

In summary, we developed a new risk prognostic model for patients with LUAD based on 8-lactate metabolism gene signatures that stratified these patients into different risk populations with different survival rates. The associations between the lactate metabolism risk score and immune infiltration, TMB, and response to ICB therapy were found to be largely consistent. These findings may contribute to the development of therapeutic strategies targeting lactate metabolism and individualized treatments for LUAD.

## Acknowledgments

We acknowledge TCGA database for providing their platforms and contributors for uploading their meaningful datasets.

## Author contributions

**Conceptualization:** Tengfei Huang, WenMin Ying.

**Data curation:** Tengfei Huang.

**Formal analysis:** Tengfei Huang.

**Funding acquisition:** WenMin Ying.

**Investigation:** Tengfei Huang, MengMeng Chen.

**Methodology:** Tengfei Huang, MengMeng Chen.

**Supervision:** WenMin Ying.

## Supplementary Material




