# Supplementary figures and images for: Prognostic value of a lactate metabolism gene signature in lung adenocarcinoma and its associations with immune checkpoint blockade therapy response
Source: Medicine (Baltimore). 2024 Oct 4;103(40):e39371. doi: 10.1097/MD.0000000000039371 (PMC11460856; doi:10.1097/MD.0000000000039371)

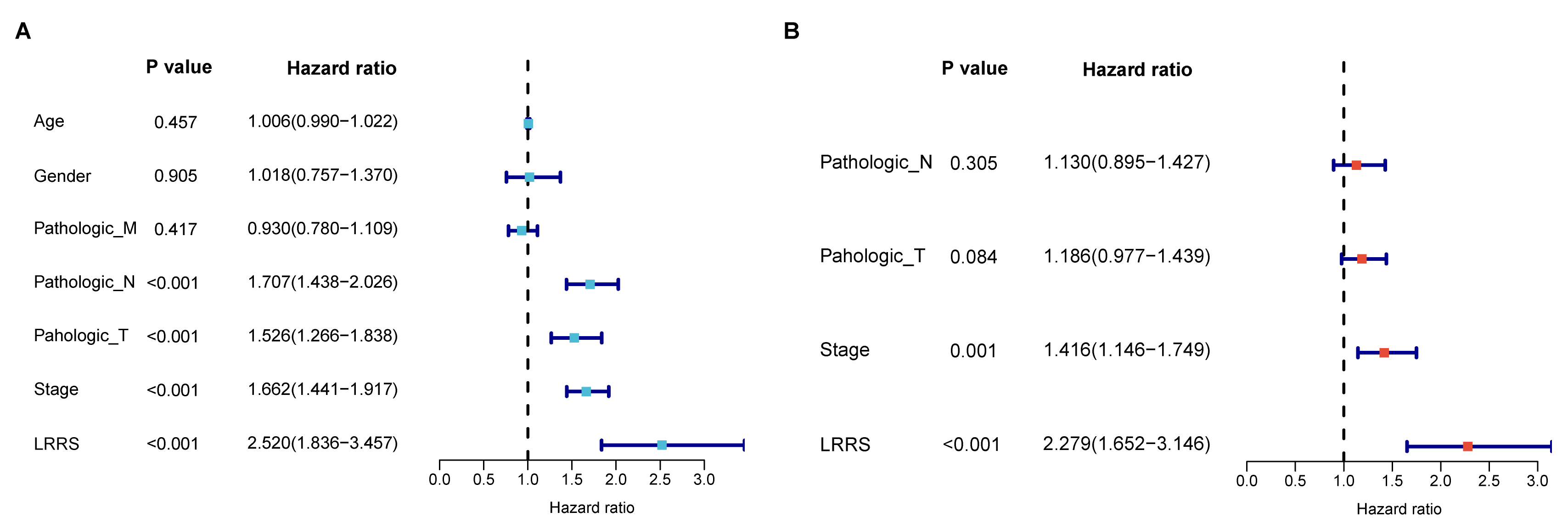

Supplement: Supplementary file 1 [file medi-103-e39371-s001.tif]

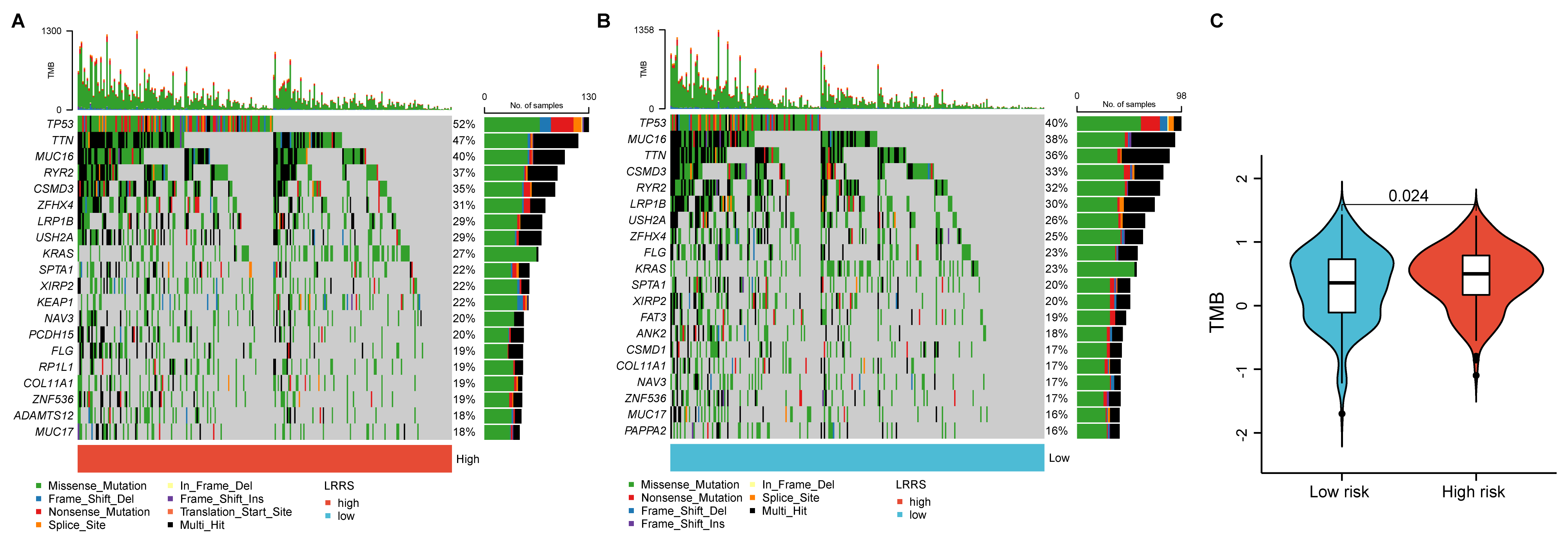

Supplement: Supplementary file 2 [file medi-103-e39371-s002.tif]
